# Effects of salinity on nest-building behaviour in a marine fish

**DOI:** 10.1186/s12898-016-0067-y

**Published:** 2016-02-29

**Authors:** Topi K. Lehtonen, Bob B. M. Wong, Charlotta Kvarnemo

**Affiliations:** Department of Biosciences, Åbo Akademi University, Tykistökatu 6, 20520 Turku, Finland; Section of Ecology, Department of Biology, University of Turku, 20014 Turku, Finland; Department of Biological and Environmental Science, University of Gothenburg, Box 463, 40530 Gothenburg, Sweden; School of Biological Sciences, Monash University, Melbourne, VIC 3800 Australia

**Keywords:** Body size, Environmental change, Nest-building, Parental care, Phenotypic plasticity, Salinity, Sand goby

## Abstract

**Background:**

Parental allocation and reproductive success are often strongly influenced by environmental factors. In this respect, salinity is a key factor influencing species distributions and community structure in aquatic animals. Nevertheless, the effects of salinity on reproductive behaviours are not well known. Here, we used the sand goby (*Pomatoschistus minutus*), a small fish inhabiting a range of different salinities, to experimentally assess the effects of changes in salinity on nesting behaviour, a key component of reproduction in sand gobies and many other taxa.

**Results:**

We found that salinity levels influenced some aspects of male nesting behaviour (i.e. nest entrance size) but not others (i.e. latency to build a nest, choice of nest site, sand on top of nest) and that small and large individuals were differently affected. In particular, the importance of body size in adjustment of nest entrance depended on the salinity level.

**Conclusion:**

The results support the prediction that geographically widespread aquatic species, such as sand gobies, are able to perform well under a range of salinity levels. The phenotype by environment interaction found between male size and behavioural responses to salinity can, in turn, help to explain the notable variation observed in nest-building (and other) behaviours closely linked to reproduction.

**Electronic supplementary material:**

The online version of this article (doi:10.1186/s12898-016-0067-y) contains supplementary material, which is available to authorized users.

## Background

Parental allocation and reproductive success are often strongly influenced by environmental factors [1]. This is especially true in species that rear their eggs or young in purpose-built nests [2–5], with nest builders often adjusting their choice of nesting site or nest architecture according to local environmental conditions [6–8]. Such adjustments, in turn, can affect the costs of nest building and nest maintenance, as well as the suitability of the conditions in the nest for the developing offspring [2, 9–13] (but see also [14]). Furthermore, nest characteristics may also act as extended phenotypic signals that reveal important information about the quality of the builder [15], with the value of this information often influenced by environmental context [16, 17].

For aquatic animals, salinity is a key factor influencing species distributions and community structure [18–21]. For instance, salinity can affect metabolic costs and growth rates of both adults and juveniles—even in species capable of surviving under a range of salinity levels [22–25]. Aside from such metabolic and physiological effects, salinity can also affect the costs and benefits of parental behaviours. For example in the flagfish, *Jordanella floridae*, salinity influences the benefits of egg-care and nest-directed behaviours [26–28]. Indeed, the effects of salinity on reproductive behaviours may be particularly pertinent in environments where salinity levels vary both spatially and temporally, such as the Baltic Sea. For instance, egg development of many marine species in the Baltic Sea is affected by the lower salinity levels [29–32], with gametes of commercially important taxa—such as cod, *Gadus morhua*, and flatfishes—being close to the limit of their salinity tolerance, while also showing local adaptation to salinity [19, 30, 33, 34].

**Fig. 1:**
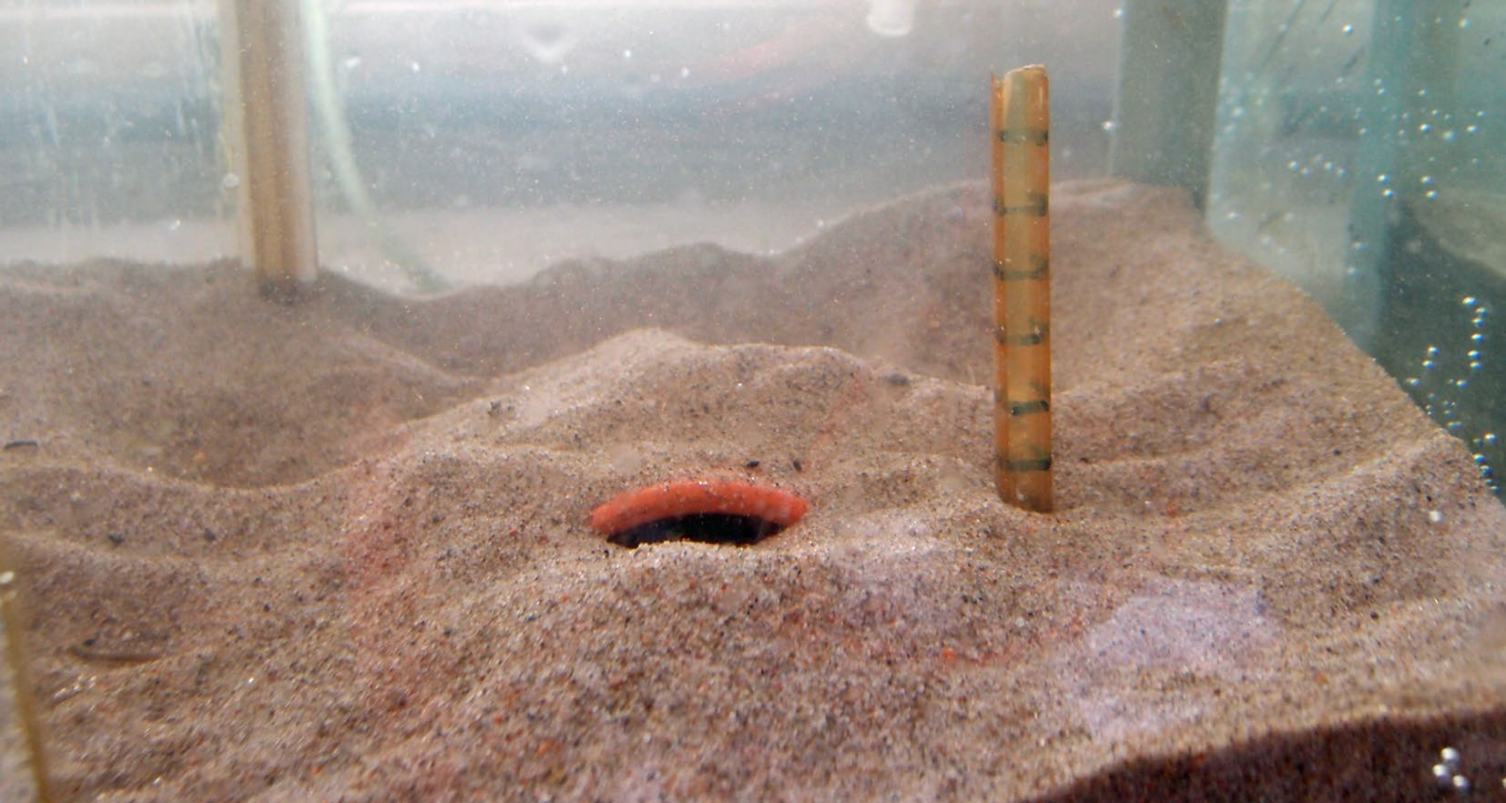
A sand goby nest constructed by piling sand on *top* of, and excavating under, a halved flowerpot

The sand goby, *Pomatoschistus minutus*, is a small marine fish with a widespread distribution across low and high salinity environments of coastal Europe, including those of the Baltic Sea [35], where salinities range from <3 ppt in the Northern Baltic to conditions close to fully marine/oceanic near the mouth of the Sea [36–39]. Male sand gobies typically build nests under empty mussel shells or rocks by excavating sand under the substrate and piling it on top of the shell or rock, leaving a single narrow opening. The size of the nesting site (also known as ‘nesting resource’ sensu [40]) and the characteristics of the nest itself can have a direct influence on male reproductive success and offspring survival. For example, nests with considerable amounts of sand on top (i.e. those that are well-covered) have been found to protect eggs and nest-tending males from predation [41]. Indeed, the amount of sand males use when building their nests can be substantial (Fig. 1), with the weight of sand piled on top of the nest sometimes exceeding 100 times the body mass of the nest-builder [40, 42]. Earlier results also suggest that food supplemented males invest more in nest building than less well-fed, control males [43]. The size of the entrance of a sand goby nest, in turn, is likely to be relevant in terms of both ventilation of the eggs and avoidance of egg predation, with ventilation being facilitated by a large nest entrance and predator defence aided by a small entrance [44, 45]. Interestingly, females prefer builders of elaborate nests in many [46–48] but not all [48–50] environmental settings. Regarding the size of the nesting resource, large nests accommodate more eggs [51, 52], while potentially also being more costly—not only to build but also to defend against nest take-overs and egg predation attempts [53, 54].

Here, we expected males to adjust both nest-building behaviour and nest architecture to key environmental conditions (see [40, 42, 44, 55]), in this case salinity levels. In particular, not only can salinity impact metabolic costs to adults and developing young, but eggs at lower salinity levels are also more susceptible to microbial infections from pathogens, such as *Saprolegnia* water moulds [56, 57]. Yet, despite the potential importance of salinity on reproductive success, very little is known about the effects of salinity on nest choice, nest-building behaviours or nest architecture. This is an important knowledge gap because we expect such behavioural adjustments to also affect the capacity of sand gobies—and other nest-building fish—to colonize new habitats and to cope with changes in salinity levels, such as those predicted to take place in the Baltic Sea [29–31].

In the current study, we experimentally assessed the effects of salinity on nest building behaviour and nest architecture in the sand goby, by focussing on a population from the low salinity environment (salinity: 5.5 ppt) of the northern Baltic Sea. We considered four mutually exclusive hypotheses. First, sand gobies are adapted to local environmental conditions. If this is the case, we predict that the latency or ability to build a nest, the size of the chosen nest site, and/or the extent of nest elaboration should differ depending on salinity, with non-native salinities being linked to costs that have a negative impact on nest building behaviour. Second, if an isotonic environment (~ 9–12 ppt) results in energy savings, as shown for some aquatic organisms [22], the nesting behaviours could, instead, be positively influenced by an intermediate salinity level. Third, because the ancestral population of sand gobies that colonised the Baltic Sea several thousand years ago lived in high salinity conditions (see [58, 59]), as do most of the modern sand goby populations outside of the Baltic Sea [38], nest building may have evolved to peak (in terms of building motivation/latency and nest elaboration) in high salinity conditions. Finally, given their geographically widespread distribution [38], it is also feasible that sand gobies may be able to perform equally well under a range of different salinity levels. In that case, we may not expect to see any differences in nesting behaviour or nest architecture.

In addition to evaluating the effects of salinity, we also assessed the relationship between the nest-related behaviours and male body size. We considered that body size may be important because recent studies have suggested that individuals with different body sizes vary in their responses to environmental conditions [40, 42, 60]. In particular, these studies found the association between male size, the level of nest elaboration and motivation to spawn successfully to be positive only in the absence of environmental disturbance, such as predation risk or water turbidity. Moreover, smaller individuals may, due to their larger surface-to-volume ratio, be less tolerant of suboptimal salinities.

## Methods

### Fish collection and housing

The study was carried out at the Tvärminne Zoological Station (59°50.7′ N; 23°15.0′ E) of the University of Helsinki in 2014 during the sand goby breeding season, which, in this population, peaks from late May to early July. With permission from the field station, we collected sand goby males for the experiment in a nature reserve located near the station and owned by the University of Helsinki. Besides using a hand trawl for catching gobies [57], we also placed artificial nesting resources (10 × 10 cm ceramic tiles) in a nearby shallow bay and waited for males to start building their nests. The nesting males were then caught using dip nets and transported to the field station. Males were first kept for a short period (less than a week) in aquaria of ~100 l, fed *ad libitum* with live mysid shrimp (*Neomysis integer*) and supplied with a continuous through flow of natural brackish seawater, pumped straight from the Baltic Sea. All stocking, acclimatisation and experimental tanks (see below) were placed in a green house that was subject to natural day/night rhythm (with the length of the day being on average 18.5 h during the time of the study).

### Acclimatisation

Before the experiment, focal males were acclimatised to the appropriate salinity treatments, i.e. 6, 12 and 24 ppt (see below). To achieve this, we haphazardly distributed male gobies into acclimatisation tanks (length × width × height of water level: 70 × 25 cm × approx. 25 cm) containing a 2 cm layer of sand as substrate. Concurrently, we had 1–2 acclimatisation tanks per salinity treatment, each housing initially 15–25 males, with new acclimatisation runs being initiated when needed. The tanks were continuously aerated and placed within larger tanks with continuously renewed seawater to ensure that the temperature of the acclimation tanks followed natural conditions and was identical to the temperature in the stock tanks.

All of the acclimatisation tanks initially contained water that was maintained at a salinity level of 6 ppt, which was achieved using a commercial sea salt mix (‘Instant Ocean’, Spectrum Brands Inc.) added to deionised water. This salinity level is slightly higher than the salinity level experienced by gobies in this part of the Baltic (~5.5 ppt). The initial salinity levels in the acclimation tanks were then adjusted over time depending on treatment. For the ‘high salinity treatment’ (24 ppt), we gradually increased the salinity level over a 7-day period until we reached a salinity level of 24 ppt. For our ‘medium salinity treatment’ (12 ppt), salinity was increased at a similar rate, this time over a 3–4 day period, until we reached the target level of 12 ppt. For the ‘low salinity treatment’ (6 ppt), no additional salt was added to the water. To help maintain high water quality in the acclimation tanks, we performed a 50 % water change on day 7 in all of the tanks, whereby water removed from the tanks was replaced with clean water of the appropriate salinity level. Apart from this, tanks were checked regularly to ensure that any water that had been lost to evaporation was replaced with deionised water to maintain the target salinity levels in the tanks. For all three treatments, males entered the experiment (see below) 7–21 days after the start of the acclimatisation period. Here, we wanted to minimise treatment differences in the time fish spent in the target salinity prior to experimentation, while also running a similar number of replicates of each treatment at any given time. During the stocking and acclimation periods, males were fed with live mysid shrimp and frozen chironomid larvae.

### Experimental design

The aim of the experiment was to investigate whether changes in salinity levels affect investment of sand goby males into nest-building. Specifically we were interested in measuring (1) the time taken for males to begin nest building (as a measure of their motivation to nest), (2) the size of the preferred nest site, and (3) the characteristics of the nest itself (i.e. nest architecture in terms of nest elaboration and nest entrance size). Before the start of each replicate, the male was weighed using an electronic balance and its total length was measured to the nearest 0.5 mm using a measuring board with a grid scale. Each focal male was then placed into an individual experimental tank measuring 75 × 25 × 20 cm (length × width × height of water level), the bottom of which was covered with a 4 cm layer of fine sand. Each tank also contained three halved clay flowerpots for the focal male to potentially select to build his nest. These flowerpots differed in size, representing small (diameter of the mouth of the pot = 4 cm), medium (6.5 cm) and large (9.5 cm) nest sites. The three nest sites were randomly assigned to the left, right and centre of each tank, all with their entrances facing the front of the aquarium.

Water in these experimental tanks was prepared as above. Each tank was aerated by a pump, with an airstone being placed in the back of the tank behind the middle nest site. As with the acclimatisation tanks, experimental tanks were placed within a larger aquarium that was supplied with a through flow of fresh seawater to ensure that temperature was the same as the stock tanks. Males from acclimatisation tanks were only transferred to experimental tanks of matching salinities. Six replicates (n_low_ = 2; n_medium_ = 1; n_high_ = 3) were discontinued because the male showed signs of distress (e.g. erratic swimming behaviour). The experiment was successfully replicated 36 times in each treatment, with male total lengths [mean ± SE] being 52.0 ± 0.8 cm, 51.9 ± 0.9 cm, and 52.1 ± 0.9 cm, and weights 1.03 ± 0.05 grams, 1.05 ± 0.06 g, and 1.04 ± 0.05 g in the low, medium and high salinity treatments, respectively (with one missing set of body size values in the high salinity treatment due to human error).

For each replicate, males were given up to 60 h to start building a nest. During this time, tanks were checked ~7 times daily between 07:30 and 22:30 to record male behaviour, male location and any signs of nest building. A male was considered to have initiated nest-building when he had started to pile sand on top of, and excavate under, the pot [40, 61, 62]. When a male was observed to have started building a nest, the time it took to initiate nest building was recorded as the time point that was midway between the check in which the onset of nest building was observed and the previous check [63]. The male was left in the tank for another 11 h before we recorded which one of the three pots it was occupying. In many replicates, the male had, at least partially, built a nest under more than one of the three nest sites (as also observed by Japoshvili et al. [61]). In such cases, we determined the male’s principal choice being the nest site inside which it had been most often observed after the onset of nest-building.

For each constructed nest, we measured two ecologically relevant nest attributes (see [61]), namely the level of nest elaboration (measured as the amount of sand the male had displaced to cover the flower pot) and the width of the entrance to the nest. The level of nest elaboration (sensu Lehtonen and Wong [50]) was assessed by carefully lifting the flowerpot into a tray and collecting the sand that had been piled on top of the pot. Due to the shape of the flowerpots, only the sand placed directly on the ridge of the pot was collected. Our visual assessment suggests that this sand sample had a high correlation with the total amount of sand the male had placed on the nest and it was at least as reliable an estimate of the total amount of sand as, for example, the height of the sand layer on the ridge of the pot [61]. The collected sand was then dried in an oven for 36 h at 60 °C, and weighed on an electric balance [50]. The width of the nest entrance was measured by taking a digital photograph of the front of the nest using an Olympus XZ-1 digital camera, with a ruler placed next to the nest entrance as a scale (Fig. 1). This scale was later used for calibration in the image analysis software ImageJ (U.S. National Institutes of Health, Bethesda, Maryland, USA) to enable us to measure the nest entrance width.

After the experiments, most of the remaining experimental males were retained for other behavioural research or, wherever possible, gradually acclimated to local seawater conditions before being returned to the sea. In cases where re-acclimatisation was not possible due to logistical constraints, animals were euthanized (four males from the 24 ppt treatment).

### Statistical analyses

To test whether the time males spent in the experimental tank before starting to build a nest differed between treatments, we applied a Cox proportional hazards survival analysis with salinity treatment and male body mass as explanatory variables. Any males that did not commence nest-building within the allocated 60 h period were ‘right censored’ for the purpose of the survival analysis [64]. To investigate the focal males choice between small, medium and large nesting sites, we used an ordinal logistic regression, again with treatment and body mass as explanatory variables. Finally, the amount of sand on the top of the nest site, as well as the nest entrance width (both log-transformed for improved normality), were analysed in two separate linear models, with salinity and male body mass as fixed effects, and the size of the chosen nest site and the time until the onset of nest building (in hours, log-transformed) as covariates. We then simplified the models by removing the salinity × body size interaction, if it was found to be non-significant [65]. All analyses were run using R 3.1.0 software (R Development Core Team).

## Results

### Time to nest building

Of the 36 males in each treatment, 34, 33 and 33 built a nest within 60 h in the low, medium and high salinity treatments, respectively, indicating no difference between the salinity treatments (G test of independence with Williams’ correction, G = 0.0102, df = 2, p = 0.99). Similarly, neither salinity (Cox proportional hazards model, salinity treatment effect, χ^2^ = 3.111, df = 2, p = 0.21), male weight (χ^2^ = 1.403, df = 1, p = 0.24), nor their interaction (χ^2^ = 1.356, df = 2, p = 0.51) had a significant effect on time from the beginning of the experiment until the onset of nest building. It is worth noting that the main effects were also non-significant in a model fitted without the interaction term.

### Choice of the size of nest site

**Fig. 2:**
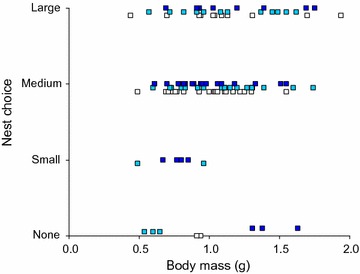
Choice of nesting site size in relation to salinity treatment and male body mass. *White boxes* low salinity, *light blue boxes* medium salinity, *dark blue boxes* high salinity

In the low salinity (6 ppt), none of the males chose the small pot, 23 chose the medium-sized pot, and 11 the large pot. In the medium salinity (12 ppt), the numbers of chosen pots were 2, 19 and 12 for small, medium and large pots, respectively. Finally, in high salinity (24 ppt), males chose 5, 19 and 9 small, medium, and large pots, respectively. Hence, the treatments did not significantly differ in the distribution of chosen nest sites (ordinal logistic regression, z = 0.721, df = 2, p = 0.47). However, male body mass had a significant effect (ordinal logistic regression, z = 2.58, p < 0.01), with larger males choosing larger nest sites (Fig. 2). Overall, males showed a strong preference towards medium-sized nest sites (61 medium nests chosen in 100 trials; binomial distribution with the H_0_ being that a medium-sized nest would be chosen every third time, p < 0.001), indifference towards large nest sites (32 large nests chosen out of 100 trials; binomial distribution, p = 0.87) and avoidance of small nest sites (7 small nest chosen; binomial distribution, p < 0.001).

### Nest characteristics

**Fig. 3:**
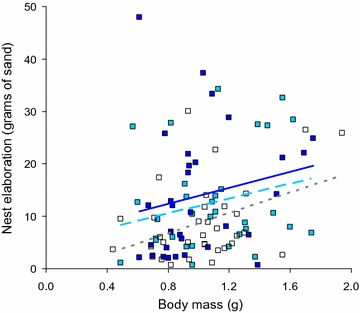
Degree of nest elaboration, measured as grams of sand on ridge of nesting site, relative to salinity and male body mass. *White boxes* + *dotted trend line* = low salinity, *light blue boxes* + *dashed trend line* = medium salinity, *dark blue boxes* + *solid trend line* = high salinity

For the amount of sand piled on top of their preferred nest site, the interaction between salinity and body mass was found to be non-significant (general linear model, F_2,90_ = 0.2947, p = 0.75) and we refitted the model without the interaction term. The simplified model showed that salinity level did not have a significant effect (F_2,92_ = 1.306, p = 0.28), whereas male body mass did (F_1,92_ = 21.64, p < 0.001). Specifically, the amount of sand piled on top of the nest was positively associated with male size (Fig. 3). Similarly, both of the covariates were significant: nests with a later onset had less sand piled on top of them (F_1,92_ = 16.41, p < 0.001) and more sand was piled on larger nests (F_2,92_ = 10.26, p < 0.001). Nevertheless, there was notable overlap in the amount of sand on the ridge of different sized nests (small: mean = 1.8 g, range 0.2–12.1 g, n = 7; medium: mean = 5.8 g, range 0.7–33.3 g, n = 61; large: mean = 13.7 g, range 0.2–28.9 g, n = 32).

**Fig. 4:**
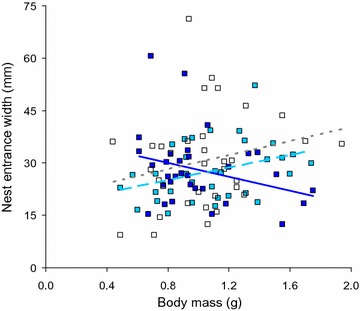
Nest entrance width in relation to salinity and male body mass. *White boxes* + *dotted trend line* = low salinity, *light blue boxes* + *dashed trend line* = medium salinity, *dark blue boxes* + *solid trend line* = high salinity

In terms of nest entrance width, we found a significant interaction between salinity and male body mass (general linear model, F_2,90_ = 7.834, p < 0.001). In particular, nest entrance width was positively associated with male body size in low and medium salinity but negatively associated in high salinity (Fig. 4). As with the sand on the nest, both the onset time (F_1,9o_ = 5.996, p = 0.016) and the size of the chosen nest (F_2,90_ = 12.02, p < 0.001) had a significant effect, with larger entrances in nests of later onset and an extensive variation in nest entrance width for each of the three nest site sizes: (small: mean = 25 mm, range 15–33 mm, n = 7; medium: mean = 25 mm, range 9–41 mm, n = 61; large: mean = 36 mm, range 18–71 mm, n = 32).

## Discussion

We found that male sand gobies from a low salinity population took comparable amounts of time to begin nest construction, irrespective of the salinities to which they were experimentally exposed. This suggests that the motivation for males to reproduce was unaffected by the increase in salinity.

An ability to perform reproductive behaviours under a range of different salinities is concordant with the expansive geographic distribution of sand gobies across coastal Europe, which encompasses both high and low salinity habitats. Indeed, the high salinity treatment in our study is the closest to the marine conditions experienced by the ancestral population of sand gobies that colonised the Baltic Sea several thousand years ago (see [58, 59]) and which most of the modern sand goby populations (outside the Baltic Sea) presently inhabit [38]. This suggests that even though the sand goby population used in our study presently inhabits a brackish water environment, they have nevertheless retained their eagerness to reproduce under higher salinity levels.

Similarly to previous work [54, 61], we found that the choice of nest site was dependent on male size, that is, larger males preferred to nest in larger nest sites. However, male choice of nesting site was not affected by salinity. Previous studies on multiple fish species have shown that the size of the nest site can have a direct bearing on male reproductive success by, for example, acting as a physical limit to the number of eggs a male is able to receive [52, 66, 67]. Larger broods, however, are also more energetically demanding to look after e.g. because they require more fanning [68]. Because metabolic demands on both the adult and the offspring are expected to vary with salinity [22–25], we might have expected sand goby males to adjust their choice of nesting sites at different salinities in response to differences in the costs of care, e.g. by choosing smaller sites at higher salinities. However, this was not the case. One possible reason is that the costs of caring for broods is unaffected by differently sized nest sites, as found in a study where male size and brood size were kept constant [69], or that the costs of caring for differently sized broods is unaffected by different salinity levels. Alternatively, males may be able to adjust their behaviours in some other way to counter any associated changes in the cost of care (e.g. through subsequent changes to parental behaviours). If either hypothesis is true, males may not need to adjust their choice of nesting site *per se*. Another possibility is that choice of nest size in this population is adapted for a low salinity environment and that gobies, when exposed to higher salinities, are making suboptimal choices with respect to the size of their nesting site. In such a situation, inappropriate behavioural responses can cause animals to make poor nesting decisions, with potentially negative consequences to offspring fitness, as has been shown, for example, in birds [70, 71].

Increases in salinity had different effects on the two measures of nest architecture examined in our study, amount of sand piled on top of the nest and nest entrance width. The amount of piled sand was not affected by salinity level. This may not be surprising if the main reason for sand piling is to help conceal the nest against potential predators [41], since the value of having a well-constructed (i.e. concealed) nest should be important irrespective of salinity. Alternatively, the amount of sand piled on top of the nest may also act as an extension of the male’s phenotype [42, 43] by revealing important information about the quality of the nest builder to choosy females (i.e. by serving as an extended phenotypic signal; sensu Schaedelin and Taborsky [15]). Hence, in the context of the current study, the degree of nest elaboration can also be important if the trait is condition dependent and if male condition is important to offspring survival. However, in a recent study, Lehtonen and Wong [50] found that male condition may be a poor predictor of hatching success in this particular sand goby population. Moreover, that same study revealed that the degree of nest elaboration and male condition was temporally unstable, making the amount of sand piled on top of the nest a potentially unreliable signal of male quality.

In contrast to the results we observed for the degree of nest elaboration, we found that the salinity treatment—in interaction with other factors—did affect nest entrance size. The size of the nest entrance is likely to be relevant to the nest holder’s fitness in a wide range of taxa [72], as recently shown for instance in birds [73, 74]. In fish, nest entrance size has been linked to offspring survival, with studies showing that larger nest entrances improve the flow of oxygenated water into the nest, which is critical to embryo development [10, 11]. Nests with larger entrances, however, are also more difficult to defend against predators and nest challengers. Not surprisingly, male sand gobies are known to adjust the size of their nest entrance according to changes in environmental conditions, such as oxygen levels, water temperature, and presence of egg predators [43–45]. In the current study, we found that the salinity effects on nest entrance size were dependent on the size of the male. Specifically, male size was positively associated with nest entrance width in low and medium salinities, but not in the high salinity treatment. Why might this be the case?

Individuals often adjust their behaviours in different ways depending on environmental context, with evidence that variation in individual responses could be adaptive [75, 76]. Body size, in particular, can be an important source of individual behavioural variation in a wide range of taxa [77, 78]. In sand gobies, several studies have previously reported differences in male reproductive behaviour linked to body size, with adjustment of nesting behaviour to environmental factors (e.g. water turbidity, predation risk, intrasexual competition), contingent upon the size of the male [40, 42, 60]. It has been suggested that this may be due to differences in the costs and benefits incurred by small and large males in response to different environmental conditions [42]. In this respect, small males may be less able or willing to build tighter (i.e. smaller) nest entrances in higher salinity environments due to additional costs to small males under such conditions (for example as a consequence of sensitivity to osmotic stress as a result of their higher surface to volume ratio; see [79]). Furthermore, nest entrance size (and associated rate of water flow) could also be linked to salinity-dependent fitness costs by influencing the susceptibility of eggs to infection at lower salinities (e.g. by *Saprolegnia* water moulds [56, 57]). Indeed, small and large males may differ in their ability to protect their developing eggs from infection (with large males having larger sperm duct glands [80] that produce an antimicrobial mucus, which parental males apply around the eggs [81]), to attract mates to fill their nests with eggs [17, 82], and to defend their nests against potential egg predators [40, 83]. Such factors could conceivably influence the way small and large males adjust their nest entrances in response to variation in salinity.

## Conclusion

We found that some aspects of male nesting behaviour were influenced by salinity, while others were not, and that the impacts of salinity were different for small and large individuals. In particular, although the level of salinity change did not affect the eagerness of sand gobies to commence nest building, it did influence the importance of body size in adjustment of nest entrance size, which is an important characteristic of the nest. Hence, our results in sand gobies suggest that even populations that complete their whole life cycle in a brackish water environment can retain the ability to perform normal reproduction-related behaviours under a range of salinity levels. The phenotype by environment interaction between male size and behavioural response to salinity may, in turn, help to explain the notable variation observed in nest-building (and other) behaviours closely linked to reproduction.

## Availability of supporting data

Our data are provided in the electronic supplementary materials (Additional file 1).

## Additional file

10.1186/s12898-016-0067-y Supporting data.

## Abbreviations

ppt: parts per thousand
cm: centimetres
g: grams
°C: degrees of celsius
df: degrees of freedom
